# Identification of Prognosis-Related Molecular Subgroups and Construction of a Prognostic Prediction Model Using Immune-Related Genes in Pancreatic Cancer

**DOI:** 10.1155/2022/7117014

**Published:** 2022-06-07

**Authors:** Xiang Fei, Lingming Kong, Chao Shi, Gang Wang, Chenhai Liu, Cheng Wang, Peng Liu, Xiaodong Tan

**Affiliations:** ^1^The Third Department of General Surgery, People's Hospital of China Medical University (Liaoning Provincial People's Hospital), Shenyang, China; ^2^Department of General Surgery, The First Affiliated Hospital of USTC, Division of Life Sciences and Medicine, University of Science and Technology of China, Hefei, China; ^3^Department of Day Surgery Ward, The First Affiliated Hospital of Harbin Medical University, Harbin, China; ^4^Department of General Surgery, Shengjing Hospital of China Medical University, Shenyang, China

## Abstract

**Background:**

Pancreatic cancer patients with similar clinicopathological status exhibit substantially different therapeutic responses, which might be caused by the vast molecular heterogeneity of tumors. In this study, we attempted to identify specific molecular subgroups and construct a prognostic prediction model based on the expression level of immune-related genes in pancreatic cancer. The transcriptome profiling, single nucleotide variation, copy number variation, clinicopathological information, and follow-up data of pancreatic cancer patients were obtained from The Cancer Genome Atlas database. Thereafter, the immune-related genes with prognostic significance were identified for further consensus cluster analysis. The molecular characteristics and clinicopathological information were compared between the identified subgroups, and a weighted correlation network analysis was performed to identify the hub genes associated with the subgroups. Finally, the prognostic prediction model based on immune-related genes was established using the least absolute shrinkage and selection operator (LASSO) analysis.

**Results:**

A total of 67 immune-relevant genes with prognostic significance were selected and used for the consensus cluster analysis. The total samples were divided into two groups, C1 and C2. The subgroup C1 had a significantly worse prognosis than C2, as well as lower levels of immune cell infiltration, which indicate an immunosuppressed state. The mutational rate of the cancer-related genes including *KRAS*, *TP*53, and *RNF*43 was higher in the C1 subgroup. The C1 subgroup was associated with more advanced tumor grade and T stage and with higher mortality. Using LASSO regression, we developed a prognostic prediction model based on the expression levels of 19 immune-related genes, which we validated in three external data sets. In addition, we identified four potential therapeutic and prognostic biomarkers (*TNNT1*, *KCNN4*, *SH2D3A*, and *PHLDA2*).

**Conclusion:**

We identified two novel molecular subgroups of pancreatic cancer and developed a prognostic prediction model based on the expression levels of immune-related genes, which could be used in a clinical setting and could aid in unraveling the molecular processes leading to the development of pancreatic cancer.

## 1. Introduction

Pancreatic cancer is one of the most lethal malignancies affecting the digestive system. It is characterized by an insidious onset with nonspecific early symptoms and early metastasis [1, 2]. As a result, pancreatic cancer patients often are at an advanced stage or present distant metastases when they are diagnosed. This greatly hinders surgical management, which is the most effective therapeutic approach for pancreatic cancer nowadays [3]. In addition, pancreatic cancer is associated with a high risk of relapse. Altogether, these factors contribute to the high mortality associated with this disease, which is the fourth leading cause of cancer-related deaths in America, and that has a five-year survival rate of less than 9% [4]. The traditional tumor staging methods that merely rely on basic clinicopathological information such as the tumor-node-metastasis classification or the age group in the same category individuals with substantial phenotypic differences have a considerable impact on prognosis. Therefore, traditional staging methods are not sensitive enough to provide individualized diagnosis and treatment for pancreatic cancer patients [5, 6]. In addition, classical serum tumor biomarkers such as carbohydrate antigen 19-9, carbohydrate antigen 125, or carcinoembryonic antigen perform poorly in the early diagnosis and outcome prediction of pancreatic cancer [7, 8]. Improving early diagnosis and developing new risk stratification methods will contribute to more effective management and a better prognosis for pancreatic cancer patients. With the rapid development and progress of sequencing methods, huge amounts of high-throughput data from different omics technologies are now available in public databases such as The Cancer Genome Atlas (TCGA) and Gene Expression Omnibus (GEO). Wise use of this information may help us further investigate the molecular mechanisms underlying the development of pancreatic cancer.

Previous studies have already investigated the molecular classification and the internal heterogeneity of pancreatic cancer using omics data [9, 10]. Integrating genomics data, Bailey et al. established four distinct molecular subtypes of pancreatic cancer (squamous, pancreatic progenitor, immunogenic, and aberrantly differentiated endocrine-exocrine), a new classification system that might contribute to understanding the molecular evolution of pancreatic cancer, as well as to developing new therapeutic methods [11]. Using transcriptional data, Collisson et al. distinguished three pancreatic ductal adenocarcinoma subtypes (classical, quasimesenchymal, and exocrine-like) that are associated with different survival and progress rates, as well as distinct therapeutic responses [12]. Finally, a classifier constructed using the expression levels of 19 miRNAs was reported to accurately predict the prognosis of pancreatic cancer patients with high sensitivity [13]. These studies highlight the potential ways in which the use of omics data can help molecularly characterize tumors, contributing to the discovery of new diagnostic and prognostic biomarkers.

The malignant behaviors of pancreatic cancer largely depend on the complex cross-talk between tumor cells and the tumor immune microenvironment [14]. A deeper understanding of the immune landscape in pancreatic cancer could help elucidate these interactions and develop new immunotherapy approaches [15]. A previous study identified four molecular subtypes of prostate cancer that markedly differed in their prognosis by analyzing the expression levels of immune-related genes. Six of these immune-related genes were used to develop a prognostic prediction model using least absolute shrinkage and selection operator (LASSO) regression [16]. To our knowledge, no molecular subgroups of pancreatic cancer have been identified using immune-related genes to date.

In this study, we attempted to identify specific molecular subtypes of pancreatic cancer that are closely associated with the immune system signature. Analyzing the expression levels of immune-related genes with prognostic value using consensus cluster analysis, we identified two specific molecular subgroups, C1 and C2. Significant differences in the overall survival (OS) and clinical features including tumor grade, T stage, and survival status were observed between the C1 and C2 subgroups. To molecularly characterize these two subgroups, we annotated the differentially expressed genes (DEGs) according to gene ontology (GO) and Kyoto Encyclopedia of Genes and Genomes (KEGG) and performed gene set enrichment analysis (GSEA). In addition, we also performed immune infiltration, mutation spectrum, and copy number variation analyses. Weighted correlation network analysis (WGCNA) identified four hub genes associated with the molecular subgroups. In the future, it is necessary to further study the function of the four genes identified, *TNNT1*, *KCNN4*, *SH2D3A*, and *PHLDA2*, and evaluate their potential use as biomarkers. Finally, we constructed a prognostic prediction model using the expression levels of 19 immune-related genes and validated it using three external data sets. In addition, uni- and multivariate Cox analyses revealed that the constructed model was an independent prognostic factor in pancreatic cancer. These results indicate that the model could be used in a clinical setting in the near future to aid clinicians in making management-related decisions.

## 2. Materials and Methods

### 2.1. Data Download and Preprocess

Transcriptome data (RNA-Seq, HTSeq-Counts type), single nucleotide variation data (MuTect2, Annotation type), copy number variation (Copy Number Segment, Masked type), clinicopathological information, and the latest follow-up data of pancreatic cancer patients from TCGA database were downloaded from the Genomic Data Commons Data Portal (https://portal.gdc.cancer.gov) on December 8, 2020 [17]. Genes with RNA-sequencing missing values in more than half of the total samples and data from patients with overall survival (OS) of less than 30 days were excluded from the subsequent analyses. A total of 171 pancreatic cancer samples met the selection criteria and were used for consensus cluster analysis. In addition, three GEO data sets, GSE62452 [18], GSE71729 [19], and GSE78229 [20], were used for external validation [21, 22]. The detailed information on pancreatic cancer samples from TCGA and the three validation data sets are provided in Supplementary Tables 1 and 2, respectively.

### 2.2. Identification of Prognosis-Related Molecular Subgroups Based on the Immune-Related Genes

To study the existence of distinct molecular subgroups in pancreatic cancer, we selected immune-related genes with prognostic significance for further analyses. First, 1,811 immune-relevant genes were obtained from the Immunology Database and Analysis Portal (ImmPort) website (https://www.immport.org/) [23]. Thereafter, log-rank and Cox survival analyses were performed to identify survival-relevant genes based on the gene expression data of pancreatic cancer samples from the TCGA data set. A total of 1,350 genes had associated *p*-values < 0.01 in both the log-rank and Cox analyses and were selected for further study. Immune-related genes with prognostic significance were defined as the intersection of potential prognosis-related markers and immune-related genes, and a total of 67 genes were selected. Detailed information about the selected genes is provided in Supplementary Table 3. Subsequently, consensus cluster analysis based on the above 67 genes was performed using the R package “ConsensusClusterPlus” [24]. The number of consensus clusters was set to 2, based on the package guidelines. Finally, the pancreatic cancer samples were separated into groups C1 (*n* = 103) and C2 (*n* = 68). OS and principal component analysis (PCA) between the two subgroups were performed with the R packages “survival,” “survminer,” and “DESeq2” [25–28].

### 2.3. Molecular Characteristics and Clinicopathological Information Comparisons between the Two Subgroups

To further elucidate the mechanisms underlying the two subgroups, the DEGs between the C1 and C2 subgroups were identified and selected using the criteria |Log2 Fold Change| > 1 and adjusted *p*-value < 0.05. A total of 2,698 DEGs were identified using the R package “DESeq2” [28]. Furthermore, GO, KEGG, and GSEA were performed using the R packages “clusterProfiler” and “enrichplot” to identify the related molecular signaling pathways of the specific subgroups [29, 30]. Detailed information on the DEGs is provided in Supplementary Table 4. In addition, the TIMER2.0 database was used to estimate and compare the immune infiltrate levels between the two subgroups [31–33]. Next, mutation spectrum analysis of the two subgroups was performed using the R package “maftools” [34]. The function “mafComapre” of the R package “maftools” was used to perform Fisher's test on all genes to detect differentially mutated genes between C1 and C2 subgroups. Correlation analysis between gene expression levels and copy numbers was analyzed. Genes with different frequencies of copy number variations were compared between C1 and C2 subgroups. Finally, the clinicopathological features were compared between the C1 and C2 subgroups.

### 2.4. Identification of Genes Associated with the Molecular Subgroups Using WGCNA

We performed WGCNA using the R package “WGCNA” to identify the genes associated with each molecular subgroup [35, 36]. The hub genes were selected and visualized using the software Cytoscape (version 3.8.2) [37, 38] and the website Metascape [39]. The web-based tool gene expression profiling interaction analysis (GEPIA) was used to visualize the expression levels of the hub genes in 179 pancreatic tumor samples and 171 normal tissue samples [40]. Overall survival (OS) and relapse-free survival (RFS) analyses of the selected hub genes in pancreatic cancer patients were performed based on the Kaplan-Meier Plotter website [41].

### 2.5. Construction of a Prognostic Prediction Model Based on 19 Immune-Related Genes

To aid clinicians in stratifying pancreatic cancer patients based on the risk level, we developed a prognostic predictor model based on the expression data of 67 immune-relevant genes via LASSO analysis using the R package “glmnet” according to the official recommendations [42, 43]. First, the total samples from the TCGA data set were randomly divided into the training (70% of the total sample, *n* = 120) and validation sets (30% of the total sample, *n* = 51). We developed a prognostic prediction model based on 19 immune-related genes using the training set. Detailed information on the 19 immune-related genes is provided in Table 1. A risk score was assigned to each patient in the training set, according to the following formula: Risk score=∑_*n*=1_^19^(Coefficient_*n*_*∗*Expression of gene_*n*_). Samples were categorized as high or low risk, considering the threshold of the median value of the overall risk scores. OS analysis was performed between the high- and low-risk groups. The predictive efficiency of the model was assessed by determining the receiver operating characteristic (ROC) curve and the area under the curve (AUC) using the R package “timeROC” [44]. The validation set and three external data sets (GSE62452, GSE71729, and GSE78229) were used to assess the validity of the model. The expression data of all data sets used were standardized using z-scores.

### 2.6. Uni- and Multivariate Cox Analyses of the Prognostic Factors

We performed univariate analysis using the constructed model and common clinicopathological factors such as age, gender, tumor grade, clinical stage, T stage, M stage, and N stage, to identify the prognosis-related factors (*p* < 0.05). Next, these significantly prognostic factors were extracted for further multivariate Cox analysis to identify the independent prognostic factors (*p* < 0.05) in pancreatic cancer.

### 2.7. Statistical Analysis

In this study, the R (version 3.6.3) and RStudio software were utilized to carry out the statistical analysis and figure preparation. *p*-values less than 0.05 were defined as statistically significant.

## 3. Results

### 3.1. Identification of Specific Molecular Subgroups Based on Immune-Related Genes Using Consensus Cluster Analysis

**Figure 1:**
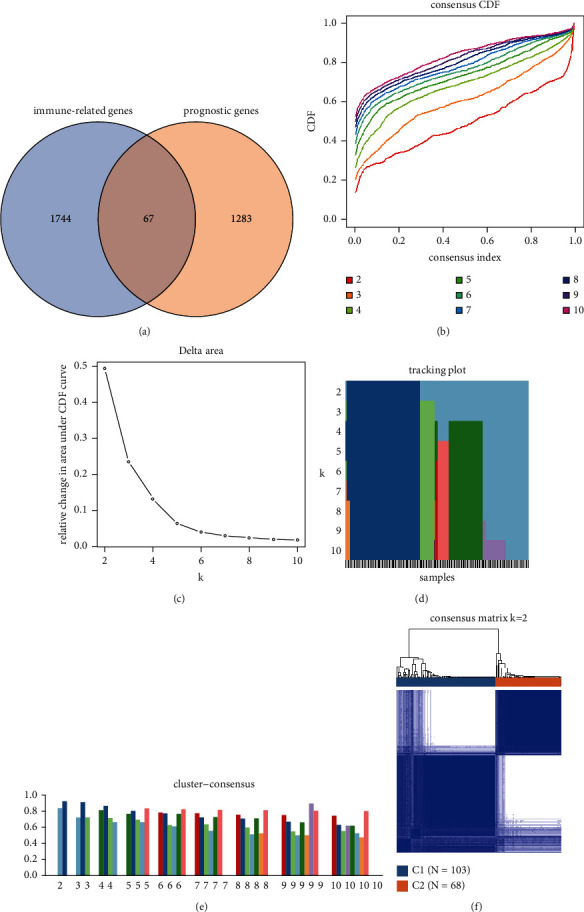
Identification of molecular subgroups using consensus cluster analysis. (a) Venn diagram that shows the intersection of the immune-related genes and the prognostic genes in pancreatic cancer. (b) Consensus cumulative distribution function (CDF) plot when the *k* value ranges from 2 to 10. (c) Relationship between the relative change in area under the CDF curve and a different number of clusters (*k* value). (d) Tracking plot of the total samples when the *k* value ranges from 2 to 10. Each color represents different clusters. (e) Relationship between the average value of the consensus matrix and each cluster when *k* value ranges from 2 to 10. The *Y*-axis stands for the average value of the consensus matrix and *X*-axis means the different number of clusters. (f) Heat map of consensus matrix when the total samples are divided into two groups, that is, C1 and C2 subgroups.

**Figure 2:**
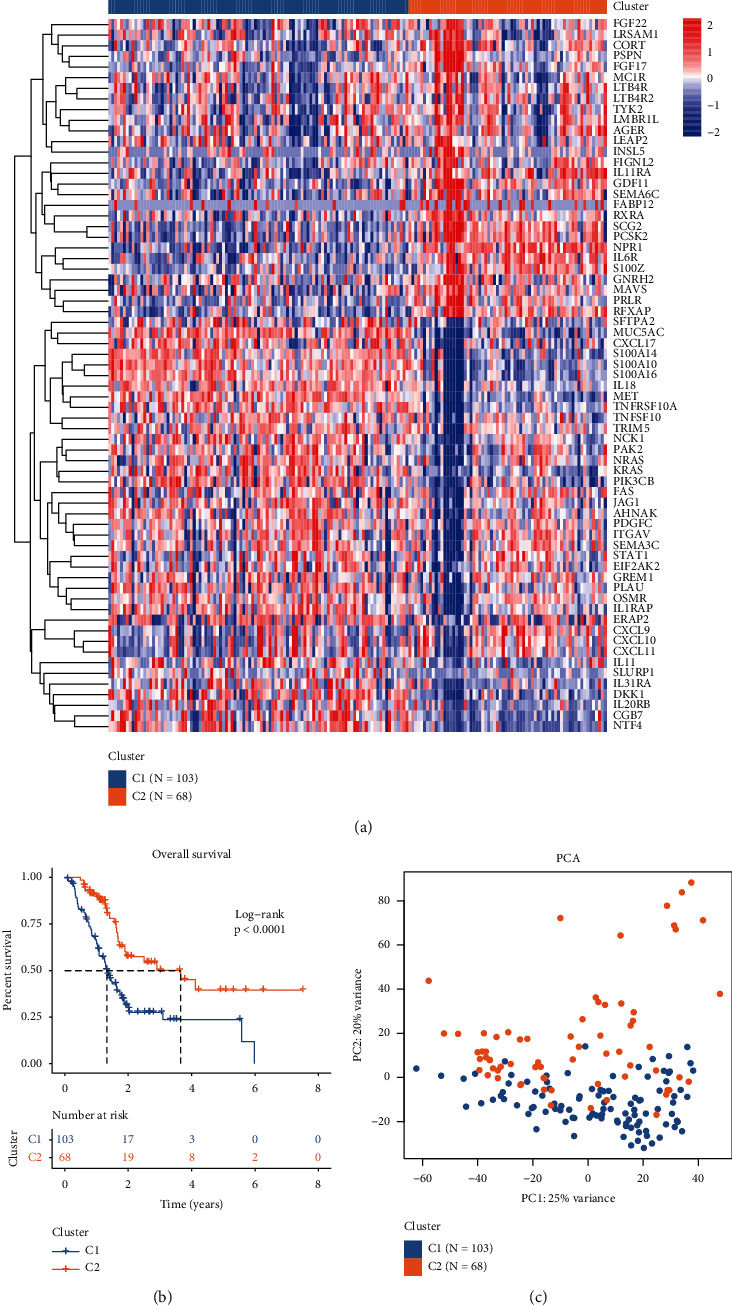
Overall survival and principal component analysis (PCA) of the C1 and C2 subgroups: (a) heat map of the expression level of the 67 immune-related genes used to identify molecular subgroups, (b) overall survival analysis between the C1 and C2 subgroups, and (c) PCA plot of the C1 and C2 subgroups.

To identify the molecular subgroups associated with immune-relevant genes in pancreatic cancer, we extracted 1,811 immune-relevant genes from the ImmPort website, which provides user-friendly bioinformatic analysis tools for basic and clinical immunology. Thereafter, log-rank and Cox survival analyses were performed to identify survival-relevant genes based on the gene expression data of pancreatic cancer samples from the TCGA data set. The genes with *p* < 0.01 in both log-rank and Cox analyses were selected and defined as genes with a prognostic value. Immune-related genes with prognostic significance were defined as the intersection of potential prognosis-related markers and immune-related genes, and a total of 67 genes were selected (Figure 1(a)). Expression data of the immune-related genes with prognostic significance were selected for further consensus cluster analysis. Samples of patients with survival time inferior to 30 days were excluded for further analyses to avoid possible disturbances. The consensus cumulative distribution function (CDF) plot showed that the slope of the CDF curve changes the most when the consensus index is set at 0 or 1 and the number of clusters is set as 2, as per official recommendations (Figure 1(b)). The relative change in the area under the CDF curve also indicated no substantial changes occur when the *k* value is higher than 2 (Figure 1(c)). A tracking plot was constructed to reflect the distribution of the samples when *k* ranges from 2 to 10 (Figure 1(d)). According to the cluster-consensus and consensus matrix plots, the total sample is divided into two distinct subgroups when *k* = 2 (Figures 1(e) and 1(f)). The pancreatic cancer samples were divided into groups C1 (*n* = 103) and C2 (*n* = 68). The expression level of the 67 immune-related genes among C1 and C2 subgroups was represented in a heat map (Figure 2(a)). The subgroup C1 has a significantly worse prognosis in comparison to the subgroup C2 (Figure 2(b); *p* < 0.0001). The existence of two distinct subgroups was further confirmed via PCA (Figure 2(c)). These results indicate the existence of two distinct molecular subgroups in pancreatic cancer according to the expression levels of prognosis-associated immune genes.

### 3.2. Identification of Signaling Pathways Related to the Molecular Subgroups

**Figure 3:**
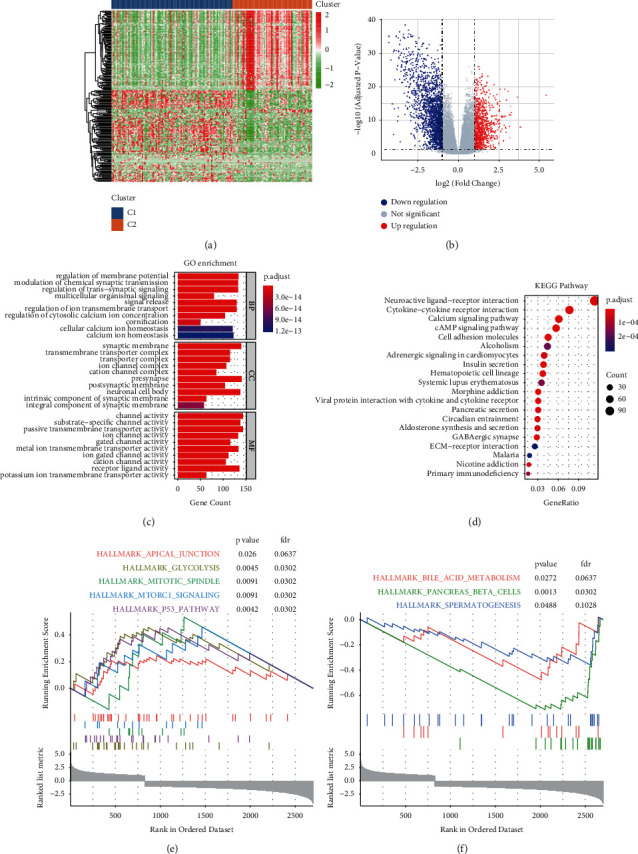
GO, KEGG, and GSEA based on the differentially expressed genes (DEGs) between the C1 and C2 subgroups: (a) heat map of the DEGs between the C1 and C2 subgroups; (b) volcano plot of the DEGs and genes with |Log2 Fold Change| > 1 and adjusted *p*-value < 0.05 were identified as significantly differentially expressed; (c) top 10 enriched items of the GO analysis including the biological process, cellular component, and molecular function; (d) top 20 signaling pathways of the KEGG enrichment analysis; (e) significantly activated signal pathways identified via GSEA; and (f) significantly inhibited signal pathways identified via GSEA.

Since the molecular subgroups of pancreatic cancer samples were significantly correlated to the OS, the DEGs between the two subgroups might be related with the initiation and development of pancreatic cancer. Therefore, we first determined the DEGs between the C1 and C2 subgroups, using the standard criteria |Log_2_ Fold Change| > 1 and adjusted *p*-value < 0.05. A total of 2,698 DEGs were identified, of which 827 were upregulated and 1,871 were downregulated (Figures 3(a) and 3(b)). To determine the function of the DEGs, GO, KEGG, and GSEA were used. The top 10 enriched terms of the GO analysis including the biological process, cellular component, and molecular function are shown in Figure 3(c) (Supplementary Table 5). Several important molecular mechanisms were enriched in the GO analysis, including regulation of trans-synaptic signaling, multicellular organismal signaling, signal release, synaptic membrane, postsynaptic membrane, ion channel activity, ion gated channel activity, and potassium ion transmembrane transporter activity. These results indicate that there is a close relationship between cellular signal transduction pathways and the molecular subgroups. KEGG enrichment analysis revealed several molecular pathways that may play a vital role in the development of pancreatic cancer, such as cytokine-cytokine receptor interaction, cAMP signaling pathway, cell adhesion molecules, pancreatic secretion, and primary immunodeficiency (Figure 3(d) and Supplementary Table 6). GSEA showed that apical junction, glycolysis, mitotic spindle, mTORC1 signaling, and p53 pathway were significantly activated, whereas bile acid metabolism and pancreas *β* cell pathways were significantly inhibited (Figures 3(e) and 3(f)). Further study of these alterations might contribute to a better understanding of the distinct molecular mechanisms underlying the two molecular subgroups identified.

### 3.3. Comparisons of Immune Infiltration, Mutation Spectrum, Clinical Features, and Copy Number Variation between the C1 and C2 Subgroups

**Figure 4:**
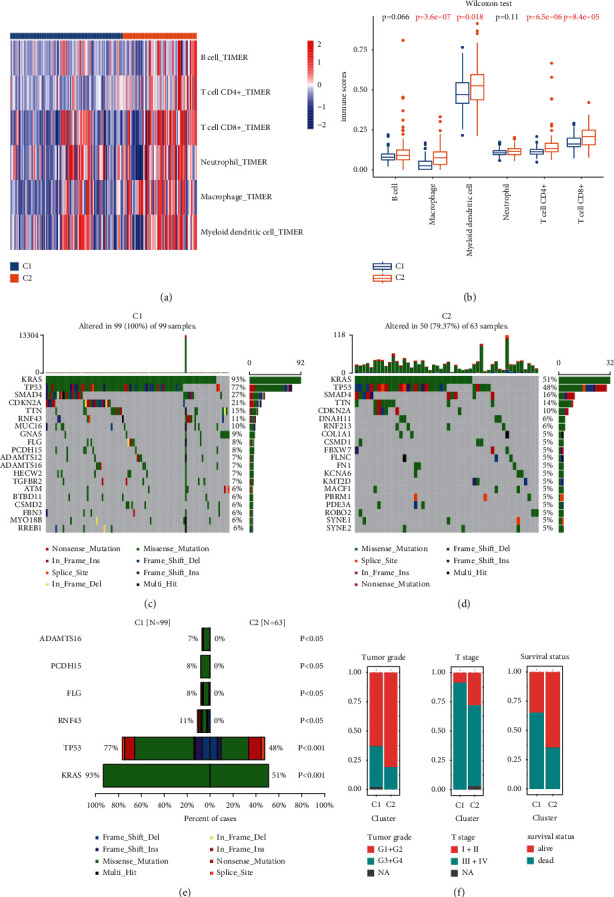
Comparisons of the immune infiltration, mutation spectrum, and clinical features between the C1 and C2 subgroups: (a) heat map of the immune infiltration levels of six types of immune cells between the C1 and C2 subgroups, (b) boxplot of the immune infiltration levels of the six types of immune cells between the C1 and C2 subgroups, (c) mutation spectrum of the top 20 genes in the C1 subgroup, (d) mutation spectrum of the top 20 genes in the C2 subgroup, (e) top six genes with different mutational frequencies between the C1 and C2 subgroups, and (f) distribution plots of different clinical features including tumor grade, T stage, and survival status between the C1 and C2 subgroups.

**Figure 5:**
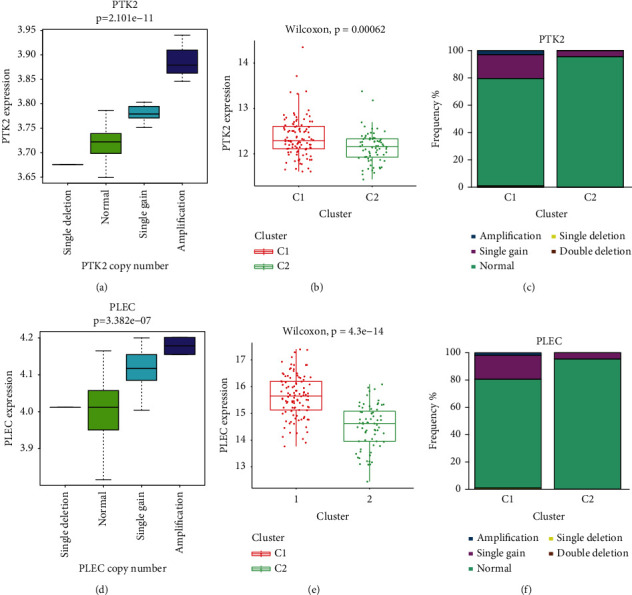
Comparisons of genes with copy number variation between the C1 and C2 subgroups: (a) correlation analysis between PTK2 gene expression level and its copy number variation, (b) PTK2 gene expression level between C1 and C2 subgroups, (c) frequency of copy number variation of PTK2 gene between C1 and C2 subgroups, (d) correlation analysis between PLEC gene expression level and its copy number variation, (e) PLEC gene expression level between C1 and C2 subgroups, and (f) frequency of copy number variation of PLEC gene between C1 and C2 subgroups.

As the subgroups identified based on the immune-related genes were significantly associated with prognosis, we compared the molecular characteristics and some clinical features between the C1 and C2 subgroups. The infiltration level of six immune-cell populations was higher in the C2 subgroup compared with the C1 subgroup (Figure 4(a)). The difference was significant for the infiltration level of macrophages, myeloid dendritic cells, T cells CD4+, and T cells CD8+ (Figure 4(b)). These results indicate an immunosuppression state in the C1 subgroup, which might contribute to the poor prognosis associated with this group. Mutational spectrum analysis of the two subgroups was performed. The top 20 mutated genes in the C1 and C2 subgroups are shown in Figures 4(c) and 4(d), respectively. The mutational frequency of *KRAS*, *T753*, *RNF43*, *FLG*, *PCDH15*, and *ADAMTS16* was significantly higher in the C1 subgroup compared with the C2 subgroup (Supplementary Table 7; Figure 4(e)). A comparison of common clinicopathological characteristics indicated that the two subgroups significantly differed in tumor grade, T stage, and survival (Table 2). The C1 subgroup, with a worse prognosis, was associated with a more advanced tumor grade and T stage and higher mortality than the C2 subgroup (Figure 4(f)). Further analyses of genes with the difference in copy number variation were performed between the C1 and C2 subgroups. Interestingly, our results demonstrated that PTK2 and PLEC expression levels were significantly correlated with their copy number (Figures 5(a) and 5(d)). The PTK2, also known as FAK, upregulation of its expression could accelerate progression and contribute to an immunosuppressive environment of pancreatic cancer [45, 46]. PTK2 gene expression was higher in the C1 than the C2 subgroup (Figure 5(b)). And the frequencies of amplification and single gain were also higher in the C1 subgroup (Figure 5(c)). In addition, previous research proved that the PLEC gene could serve as a novel biomarker to identify preinvasive, primary, and metastatic pancreatic ductal adenocarcinoma, and its expression was continuously increasing along with tumor progression [47]. Similarly, PLEC expression was also significantly higher in the C1 subgroup compared with the C2 subgroup, and frequencies of amplification and single gain were lower in the C2 subgroup than the C1 subgroup (Figures 5(e) and 5(f)). The above results might contribute to elucidating the underlying mechanisms behind the heterogeneity between different molecular subgroups.

### 3.4. Hub Genes Associated with the Molecular Subgroups Were Identified via WGCNA

**Figure 6:**
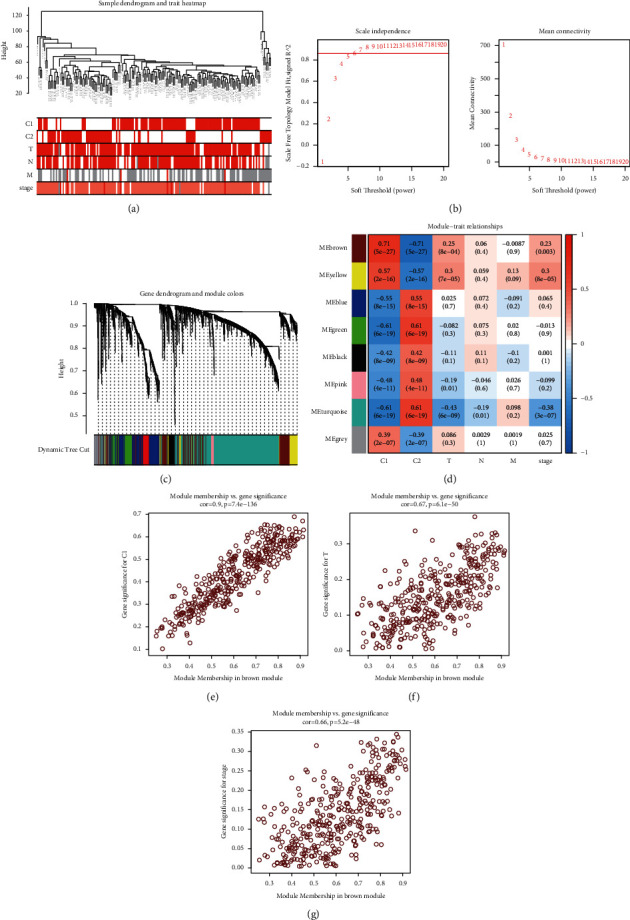
Identification of genes associated with the molecular subgroups using WGCNA: (a) dendrogram of total samples and heat map of clinical traits, (b) correlation between the soft threshold and scale-free topology model fit (left plot) and correlation between the soft threshold and mean connectivity (right plot), (c) gene dendrogram and modules in different colors, (d) correlation analysis between the gene modules and clinical traits, (e) correlation analysis between module membership in brown module and gene significance for C1 subgroup, (f) correlation analysis between module membership in brown module and gene significance for T stage, and (g) correlation analysis between module membership in brown module and gene significance for the clinical stage.

**Figure 7:**
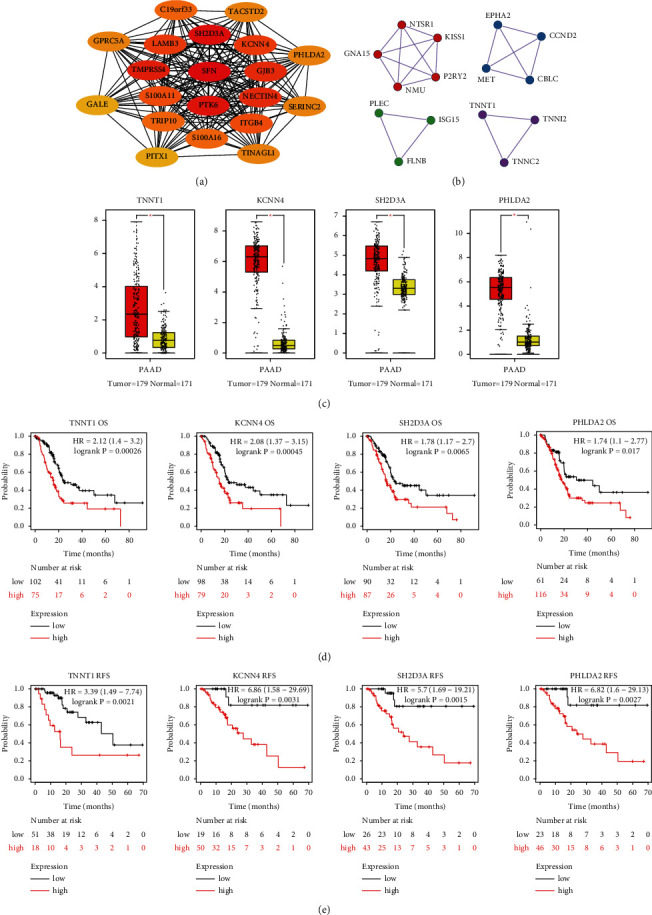
Identification of four potential target genes in pancreatic cancer: (a) top 20 hub genes of the brown module are selected by using the Cytoscape software; (b) hub genes of the brown module are selected by using the metascape database; (c) expression levels of *TNNT1*, *KCNN4*, *SH2D3A*, and *PHLDA2* between the 179 pancreatic tumors and 171 normal tissue samples based on the GEPIA database; (d) overall survival analysis of *TNNT1*, *KCNN4*, *SH2D3A*, and *PHLDA2* in pancreatic cancer using the Kaplan-Meier Plotter website; and (e) relapse-free survival of TNNT1, *KCNN4*, *SH2D3A*, and *PHLDA2* in pancreatic cancer using the Kaplan-Meier Plotter website.

We performed WGCNA of 2,698 DEGs to identify specific gene coexpression modules, and subsequently, we identified the gene coexpression module most correlated with the clinical traits. The complete clinical information is shown in the clustering dendrogram with the trait heat map (Figure 6(a)). The soft threshold of WGCNA was defined as 6 to maintain the balance between scale independence and mean connectivity (Figure 6(b)). Genes were separated into eight modules depicted in different colors in Figure 6(c). The correlation analysis between the gene modules and clinical traits identified that the module labeled as brown was the one most significantly correlated with the C1 subgroup (Figure 6(d)). In addition, this module was also significantly correlated with the T stage and the clinical stage. Gene significance, defined as the correlation between gene expression and clinical traits, was put in relation to module membership, defined as the correlation between the module and the gene expression profile. The correlation between module membership and gene significance for the T stage, the clinical stage, or the subgroup in the brown module is shown in Figures 6(e)–6(g). Detailed information on the genes in the brown module is provided in Supplementary Table 8. To further identify the hub genes in the brown module, the hub genes were selected using the software Cytoscape (Figure 7(a)) and identified using the database Metascape (Figure 7(b)). Analysis of the expression level of the hub genes indicated that the genes *TNNT1*, *KCNN4*, *SH2D3A*, and *PHLDA2* were differentially expressed between 179 pancreatic tumors and 171 normal tissue samples (Figure 7(c)). The expression level of the four genes was significantly correlated with the OS, with higher expression levels indicating a worse prognosis (*p* < 0.05; Figure 7(d)), and with RFS (*p* < 0.05; Figure 7(e)). These results suggest that these four genes might play an important role in the progression of pancreatic cancer. Further *in vitro* and *in vivo* experiments should be performed to verify its biological function in pancreatic cancer.

### 3.5. Construction of the Prognostic Prediction Model Based on the 19 Immune-Related Genes

**Figure 8:**
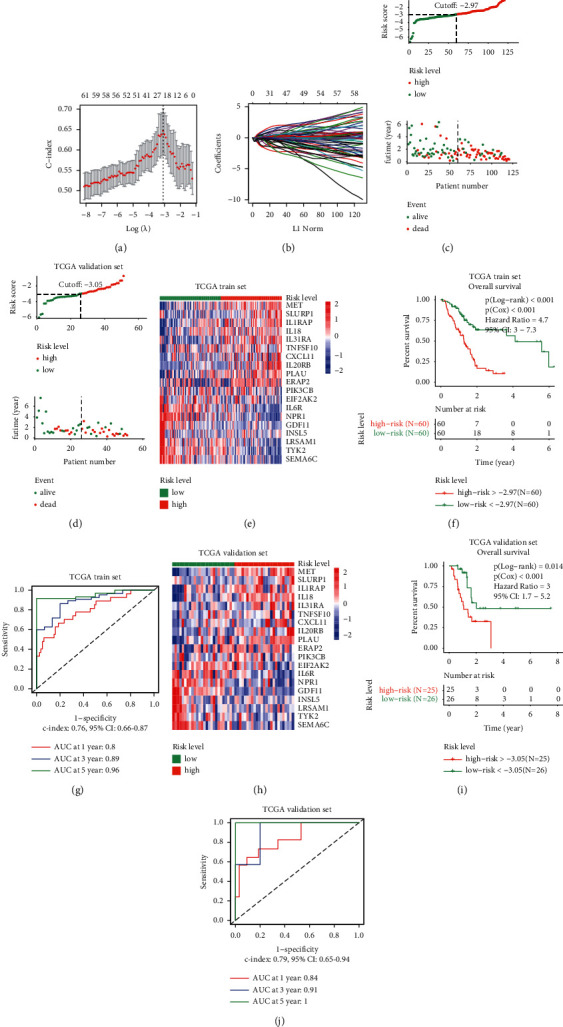
Construction and validation of the prognostic prediction model based on the 19 immune-related genes: (a) relationship between c-index and log (*λ*) value in the LASSO regression model, (b) coefficients of different genes corresponding to different combination models, (c) distribution plots of risk score and survival time between the high- and low-risk groups in the TCGA training set, (d) distribution plots of risk score and survival time between the high- and low-risk groups in the TCGA validation set, (e) heat map of 19 genes in the TCGA training set, (f) overall survival (OS) analysis between the high- and low-risk groups in the TCGA training set, (g) the ROC curve was used to evaluate the efficiency of the 19 gene model in the TCGA training set, (h) heat map of 19 genes in the TCGA validation set, (i) OS analysis between the high- and low-risk groups in the TCGA validation set, and (j) the ROC curve was used to evaluate the efficiency of the 19 gene model in the TCGA validation set.

In this study, a prognostic prediction model was developed to accurately stratify pancreatic cancer patients according to the risk level, which could be a tool of great importance in a clinical setting. To select the genes to construct the model, LASSO regression was performed using the 67 immune-relevant genes with prognostic significance. Samples in the TCGA data set were randomly divided into the training (70% of the total samples, *n* = 120) and validation sets (30% of the total samples, *n* = 51). The prognostic prediction model was constructed using the training data set using the LASSO regression model. The c-index was highest when log (*λ*) = −3.08 (Figure 8(a)). With these parameter values, a total of 19 immune-related genes were selected to construct the model. The coefficients of different genes corresponding to various combination models are shown in Figure 8(b). Detailed information on 19 genes and their coefficients is listed in Table 1. A risk score was calculated for each patient according to the following formula:

(1) $$\begin{array}{c} \text{Risk score}=\sum_{n=1}^{19}\left({\text{Coefficient}}_{n}*\text{Expression of }{\text{gene}}_{n}\right). \end{array}$$

The distribution plots of risk score and OS between the high- and low-risk groups in the training set are shown in Figure 8(c). A heat map of the expression levels of the selected genes in the training set is depicted in Figure 8(e). The OS analysis between the high-risk (*n* = 60) and low-risk (*n* = 60) groups showed that the high-risk group had a significantly worse prognosis (log-rank *p* < 0.001, Cox *p* < 0.001, HR = 4.7, 95% CI: 3 − 7.3; Figure 8(f)). The efficiency of the model constructed based on the expression levels of the 19 genes was assessed using a ROC curve. The AUC of 1-, 3-, and 5-year survival time were 0.80, 0.89, and 0.96, respectively (Figure 8(g), c-index: 0.76, 95% CI: 0.66–0.87). Thereafter, the samples in the validation set were classified as high or low risk based on the median value of the overall risk scores. The risk score and OS distribution plots and the heat map of the genes for the internal validation set are provided in Figures 8(d) and 8(h), respectively. A significant difference between the high-risk (*n* = 25) and low-risk (*n* = 26) groups was also identified in the internal validation set (log-rank *p* < 0.014, Cox *p* < 0.001, HR = 3, 95% CI: 1.7 − 5.2; Figure 8(i)). The AUC of 1, 3, and 5 years in the ROC curve were 0.84, 0.91, and 1.00 in the internal validation set, respectively (Figure 8(j), c-index: 0.79, 95% CI: 0.65–0.94). These results indicated that the model based on the expression levels of the selected immune-related genes could serve as an accurate prognostic prediction tool in pancreatic cancer.

**Figure 9:**
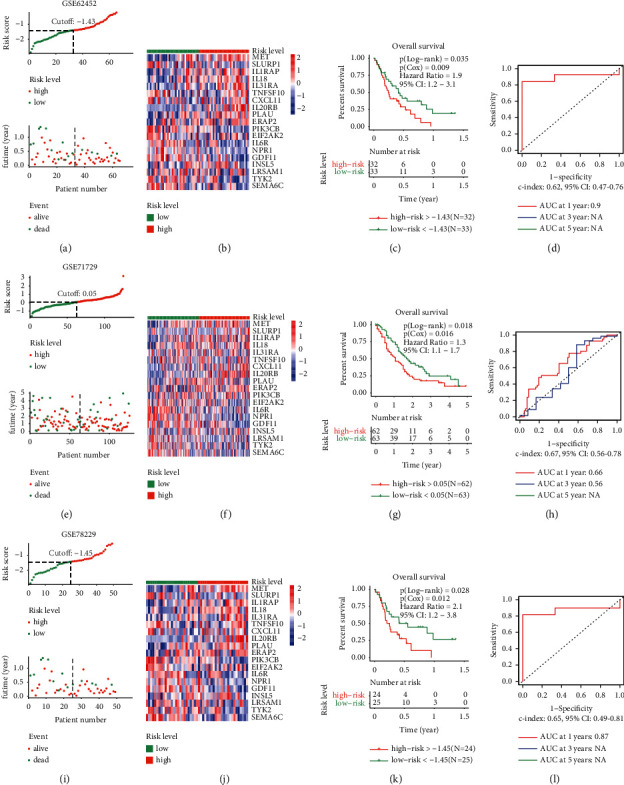
Validation of the prognostic prediction model in the three external validation sets from the GEO database: (a) distribution plots of risk score and survival time between the high- and low-risk groups in the GSE62452 data set, (b) heat map of 19 genes in the GSE62452 data set, (c) overall survival (OS) analysis between the high- and low-risk groups in the GSE62452 data set, (d) the ROC curve was used to evaluate the efficiency of the 19 gene model in the GSE62452 data set, (e) distribution plots of risk score and survival time between the high- and low-risk groups in the GSE71729 data set, (f) heat map of 19 genes in the GSE71729 data set, (g) OS analysis between the high- and low-risk groups in the GSE71729 data set, (h) the ROC curve was used to evaluate the efficiency of the 19 gene model in the GSE71729 data set, (i) distribution plots of risk score and survival time between the high- and low-risk groups in the GSE78229 data set, (j) heat map of 19 genes in the GSE78229 data set, (k) OS analysis between the high- and low-risk groups in the GSE78229 data set, and (l) the ROC curve was used to evaluate the efficiency of the 19 gene model in the GSE78229 data set.

The universality of the model was investigated via external validation using multiple data sets obtained from the GEO database (GSE62452, GSE71729, and GSE78229). According to the risk score formula, the samples of the three external data sets were separated into high- and low-risk groups. The risk score and survival time distribution plots in the high- and low-risk groups from the GSE62452 data set are shown in Figure 9(a), those from GSE71729 in Figure 9(e), and those from GSE78229 in Figure 9(i). The heat map of the expression levels of the 19 immune-related genes in the GSE62452, GSE71729, and GSE78229 data sets are shown in Figures 9(b), 9(f), and 9(j), respectively. OS analysis showed that the prognosis of the low-risk group was better than that of the high-risk group in the three data sets (for GSE62452, log-rank *p*=0.035, Cox *p*=0.009, HR = 1.9, 95% CI: 1.2–3.1, Figure 9(c); for GSE71729, Log-rank *p*=0.018, Cox *p*=0.016, HR = 1.3, 95% CI: 1.1–1.7, Figure 9(g); and for GSE78229, Log-rank *p*=0.028, Cox *p*=0.012, HR = 2.1, 95% CI: 1.2–3.8, Figure 9(k)). The AUC for 1-year OS in the ROC curve in GSE62452 was 0.9 (Figure 9(d), c-index: 0.62, 95% CI: 0.47–0.76). The AUC for 1- and 3-year OS in GSE71729 were 0.66 and 0.56, respectively (Figure 9(h), c-index: 0.67, 95% CI: 0.56–0.78). The AUC for 1-year OS in the GSE78229 data set was 0.87 (Figure 9(l), c-index: 0.65, 95% CI: 0.49–0.81). These results demonstrated that the prognostic prediction model based on the 19 immune-related genes also had a satisfactory function in the external validation data sets.

### 3.6. Uni- and Multivariate Cox Analyses of the Prognostic Factors

To identify the prognosis-associated factors for pancreatic cancer patients, univariate Cox regression analysis was performed on the risk score model and common clinicopathological information, including age, gender, tumor grade, clinical stage, T stage, M stage, and N stage based on the data from TCGA data set. Risk score, age, tumor grade, T stage, and N stage could serve as prognosis-related factors (*p* < 0.05). Multivariate Cox regression analysis indicated that from these five prognosis-related factors, risk score and N stage were independent prognostic predictor factors (*p* < 0.05). Detailed results of the uni- and multivariate Cox analysis are provided in Table 3. These results demonstrate that the prognostic prediction model based on the 19 immune-related genes could serve as an independent prognostic factor.

## 4. Discussion

Pancreatic cancer patients with similar tumor morphology and clinicopathological status often show considerable differences in responses to the same therapeutic method, which may be caused by the vast molecular heterogeneity of the tumor tissue [10, 48, 49]. Accurately stratifying patients in molecular subgroups with specific OS, clinical outcome, and therapeutic responses would aid clinicians in making accurate decisions and administering individualized treatment, leading to a better prognosis in pancreatic cancer [50–52]. As the immune landscape is closely associated with the development of pancreatic cancer and immunotherapy stands as a promising therapeutic option in the near future [53, 54], we attempted to identify distinct molecular subgroups in pancreatic cancer by analyzing the expression level of immune-related genes.

For this purpose, we selected the expression data of the immune-related genes with *p* < 0.01 in both log-rank and Cox survival analyses for further analysis. To identify the distinct molecular subgroups, we performed consensus cluster analysis, an unsupervised technique that allows the grouping of similar objects and division of the data and has been widely used to study the existence of distinct subgroups in various cancer types [55, 56]. Using this approach, the samples were divided into two distinct molecular subgroups, C1 (*n* = 103) and C2 (*n* = 68), which presented significant differences in the prognosis.

To further elucidate the underlying molecular mechanisms behind the established molecular subgroups, we utilized GO, KEGG, and GSEA by employing the DEGs to identify the signaling pathways specifically associated with each subtype. Immune infiltration analysis revealed that the immune scores of six immune cell types were higher in the C2 subgroup than in the C1 subgroup. These differences were significant for macrophages, myeloid dendritic cells, and the T cell populations CD4+ and CD8+. These results suggest a state of immune suppression in the C1 subgroup, which might underlie the poor prognosis associated with this subgroup. Mutation analysis indicated that classical cancer-related genes such as KRAS, TP53, and RNF43 were more frequently mutated in the C1 subgroup [57–59]. Analyses of genes with the difference in copy number variation were performed between the C1 and C2 subgroups. The expressions of the cancer-promoting PTK2 and PLEC genes were significantly higher in the C1 subgroups than the C2 subgroups. Besides, the two genes' frequencies of amplification and single gain were lower in the C2 subgroup than the C1 subgroup. Finally, analysis of the clinical features in the two subgroups revealed that a more advanced tumor grade and T stage and higher mortality were associated with the C1 subgroup. Our analysis provides a comprehensive perspective of the differential characteristics of the two identified groups.

To identify the DEGs specifically associated with each molecular subgroup, we performed WGCNA. The coexpression gene module labeled in brown in Figure 6(d) was the one most significantly correlated with the molecular subgroups. The hub genes in this module were identified using the Cytoscape software and the Metascape database. The expression level of genes *TNNT1*, *KCNN4*, *SH2D3A*, and *PHLDA2* was significantly different between the 179 pancreatic tumors and 171 normal tissue samples. In addition, the expression level of these genes was significantly correlated with the OS and RFS of pancreatic cancer patients. A previous study reported that *TNNT1* is significantly upregulated in breast tumor samples, and it facilitated their uncontrolled proliferation of tumor cells [60]. In addition, another study reported *TNNT1* overexpression in colorectal cancer cells, where it enhances their proliferation, migration, and invasion capacities [61]. *KCNN4* has been shown to modulate epithelial-mesenchymal transition and cell apoptosis, increasing the malignant behavior of papillary thyroid cancer cells [62]. Finally, high expression of *SH2D3A* has been reported to enhance the progression of ovarian cancer [63], and downregulation of *PHLDA2* has been reported to significantly inhibit the development of colorectal cancer through the PI3K/AKT signaling pathway [64]. In the future, we aim to study in detail the function of these four genes using *in vitro* and *in vivo* approaches.

We constructed a risk model using the expression levels of 19 immune-related genes using LASSO regression. The resulting model performed well in the internal training and validation TCGA subsets, as well as in three external validation data sets, although the AUC for 3- and 5-year OS were not all available in the three external data sets due to time limitations. Finally, uni- and multivariate Cox analyses demonstrated that the prognostic-prediction model developed using these genes could serve as an independent prognostic factor, indicating its potential use in a clinical setting.

## 5. Conclusions

In this study, we established two specific molecular subgroups based on the immune-related genes with prognostic significance using consensus cluster analysis. The two subgroups demonstrated significant differences in the OS and clinical features, including tumor grade, T stage, and survival status. The signaling pathways identified using GO, KEGG, and GSEA could contribute to understanding the underlying mechanisms behind the molecular classifications. The immune infiltration, copy number variation, and mutation spectrum analysis provided novel insights into the molecular subgroups. Four potential biomarkers, TNNT1, KCNN4, SH2D3A, and PHLDA2, were identified using WGCNA. The prognostic prediction model based on 19 immune-related genes could serve as an effective tool to predict the overall survival of pancreatic cancer patients.

## Figures and Tables

**Table 1:** The 19 immune-related genes and their corresponding coefficients that were selected to construct the prognostic prediction model based on the LASSO regression model.

Number	Gene name	LASSO coefficient
1	MET	0.0951
2	SLURP1	0.8368
3	IL1RAP	0.0332
4	IL18	0.3994
5	IL31RA	0.6839
6	TNFSF10	0.1371
7	CXCL11	0.4979
8	IL20RB	0.2564
9	PLAU	0.1469
10	ERAP2	0.2966
11	PIK3CB	−0.9853
12	EIF2AK2	−1.1102
13	IL6R	−0.2665
14	NPR1	−0.2134
15	GDF11	−1.2324
16	INSL5	−0.0406
17	LRSAM1	−0.6822
18	TYK2	−0.0659
19	SEMA6C	−0.3146

**Table 2:** Comparisons of clinicopathological information between the C1 and C2 subgroups in pancreatic cancer.

Clinicopathological factors	Consensus cluster		*p*-value
	C1 (*n* = 103)	C2 (*n* = 68)	
Age (year), median (IQR)	65.0 (56.5–74.0)	65.0 (57.0–71.0)	0.4298^a^
Gender			0.3495^b^
Female	44	34	
Male	59	34	
Tumor grade			0.0202^b^
G1 + G2	65	55	
G3 + G4	36	13	
NA	2	0	
Clinical stage			0.7055^c^
I + II	97	64	
III + IV	5	2	
NA	1	2	
T stage			0.0006^b^
T1 + T2	9	19	
T3 + T4	94	47	
NA	0	2	
M stage			0.6355^c^
M0	44	33	
M1	3	1	
NA	56	34	
N stage			0.8867^b^
N0	29	18	
N1	72	47	
NA	2	3	
Survival status			0.0001^b^
Alive	36	44	
Dead	67	24	

^a^The Mann–Whitney U test, ^b^chi-square test, and ^c^Fisher's exact test were used to calculate the *p*-values. IQR: Interquartile range. NA: not available.

**Table 3:** Uni- and multivariate Cox analyses of the prognostic factors in pancreatic cancer.

Prognostic factors	Univariate Cox analysis				Multivariate Cox analysis			
	HR	Lower 95% CI	Upper 95% CI	*p*-value	HR	Lower 95% CI	Upper 95% CI	*p*-value
Risk score	3.949	2.801	5.566	<0.001	3.838	2.626	5.610	<0.001
Age	1.030	1.008	1.052	0.006	1.016	0.995	1.036	0.135
Gender	0.802	0.531	1.211	0.294				
Grade	1.457	1.094	1.941	0.010	0.964	0.698	1.331	0.823
Stage	1.314	0.897	1.926	0.161				
T	1.555	1.002	2.413	0.049	0.968	0.575	1.629	0.904
M	1.028	0.246	4.297	0.970				
N	2.082	1.238	3.501	0.006	1.891	1.097	3.260	0.022

## Data Availability

All data generated or analyzed during this study are included in this published article and its supplementary information files.

## Supplementary Materials

Supplementary Materials Supplementary Tables 1–8 were uploaded to the submission system. Supplementary Table 1: Detailed information on pancreatic cancer samples from the TCGA data set. Supplementary Table 2: Detailed information on pancreatic cancer samples from the three validation data sets including GSE62452, GSE71729, and GSE78229. Supplementary Table 3: A total of 67 immune-related genes with prognostic significance (*p*-values < 0.01 in both the log-rank and Cox analyses) were selected for further analysis. Supplementary Table 4: Detailed information on the differentially expressed genes between the C1 and C2 subgroups. Supplementary Table 5: Detailed results of the gene ontology (GO) analysis including the biological process (BP), cellular component (CC), and molecular function (MF). Supplementary Table 6: Detailed results of the Kyoto Encyclopedia of Genes and Genomes (KEGG) enrichment analysis. Supplementary Table 7: Genes with the difference in the mutational frequency between the C1 and C2 subgroups. Supplementary Table 8: Detailed information on the genes in the brown module of the weighted correlation network analysis (WGCNA).
